# New views on physiological functions and regulation of butyrylcholinesterase and potential therapeutic interventions

**DOI:** 10.3389/fmolb.2025.1625318

**Published:** 2025-06-19

**Authors:** Samaneh Hajimohammadi, Oksana Lockridge, Patrick Masson

**Affiliations:** ^1^ Department of Pharmacodynamics and Toxicology, School of Pharmacy, Mashhad University of Medical Sciences, Mashhad, Iran; ^2^ Student Research Committee, Mashhad University of Medical Sciences, Mashhad, Iran; ^3^ Eppley Institute, University of Nebraska Medical Center, Omaha, NE, United States; ^4^ Laboratory of Biochemical Neuropharmacology, Kazan Federal University, Kazan, Russia

**Keywords:** acetylcholinesterase, butyrylcholinesterase, functions, microRNA or miR, organophosphate poisoning, bioscavenger

## Abstract

Butyrylcholinesterase (BChE) is widely distributed in human tissues, although its physiological roles remain incompletely defined. It contributes modestly to cholinergic transmission and participates in lipid and ghrelin metabolism. BChE is pharmacologically and toxicologically significant due to its ability to hydrolyze various esters and neutralize toxic compounds such as carbamates and organophosphate (OP) pesticides and nerve agents. This review explores current insights into BChE functions and regulatory physiological mechanisms, with particular emphasis on its interaction with microRNAs (miRNAs) and defense against toxicants. BChE serves as a bioscavenger of OPs and reversible inhibitors, including several drugs and environmental chemicals. Moreover, its plasma activity is recognized as a valuable biomarker for disease prognosis, treatment monitoring, and the assessment of OP poisoning severity. Recent findings revealed a strong connection between specific miRNAs and BChE regulation, positioning these small non-coding RNAs as potential indicators of metabolic dysfunction and diverse pathological conditions. Additionally, miRNAs appear to modulate BChE expression in response to stress, inflammation, and immune responses. These discoveries highlight the potential of miRNA-based therapeutic approaches targeting BChE pathways in various clinical settings.

## Graphical Abstract

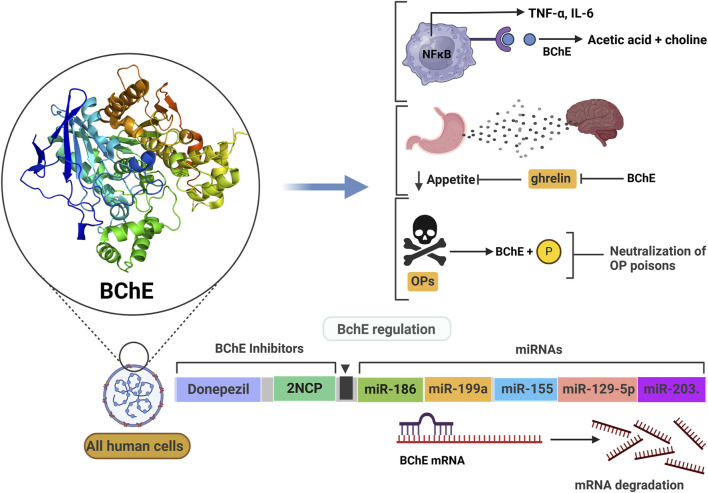

## 1 Introduction

**FIGURE 1:**
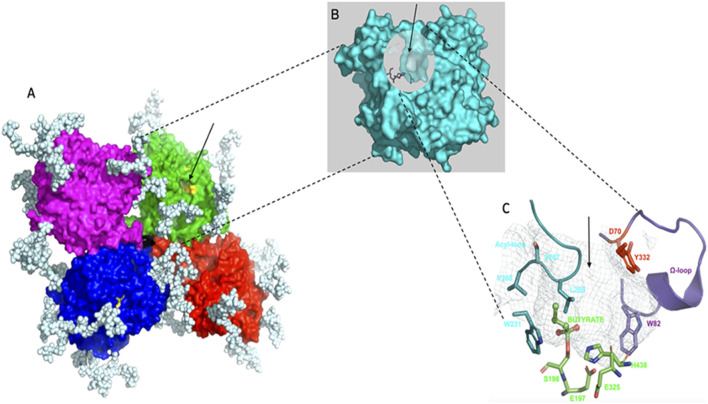
Structure of human BChE. **(A)**, tetramer, 340 kDa organized as a dimer of dimers with non-planar arrangement of identical monomers, monomers in each dimer are covalently linked through a single disulfide bridge (the white chains are complex-type glycans, 9 glycan chains per monomer); **(B)**, monomer, 85 kDa, in tetramer the 4 monomers are identical and independent, showing no cooperativity in binding and reactions, there is one active site per monomer; **(C)**, active site gorge, the arrow indicates the entrance of the gorge, the active site serine (S198) is at the bottom of the 20 Å deep gorge, the peripheral site (D70, Y332) – initial binding site of substrates and ligands - located at the entrance of the gorge is connected to the catalytic active center through an allosteric loop, the $\Omega \text{ loop }\left(\text{in violet}\right)$ , connecting D70 to W82. Residue W82 is the main binding residue of the active center.

BChE (EC. 3.1.1.8, accession number P06276) has been known for a century and extensively investigated. Yet, its physiological functions are still imperfectly known. It is structurally, catalytically and functionally related to acetylcholinesterase (AChE; EC.3.1.1.7, P22303), the enzyme that terminates the action of acetylcholine, the neurotransmitter of the cholinergic system (Silman, 2021). In humans, the BCHE gene (locus E1) is located on chromosome 3 at position 3q26.1-q26.2 (Allderdice et al., 1991). There is no alternative splicing of the gene, and the unique mRNA is encoded by 4 exons (Massoulié, 2002). However, BChE displays a considerable genetic polymorphism (polyallelism at locus E1 and heterogeneous isoenzymes depending on different loci, E2 and other E loci) (Lockridge, 2015). In addition, the existence of multiple soluble and membrane-anchored molecular forms (Massoulié, 2002; Schopfer et al., 2017) complicates molecular polymorphic patterns. Most human tissues produce the enzyme. However, plasma BChE, whose average concentration is 5 mg/L, is secreted by hepatocytes. The major form in plasma is a homo-tetramer of 340 kDa (G4). The structure of the monomer (G1) was solved by x-ray crystallography PDB 1P0I (Nicolet et al., 2003) and the structure of the tetramer was later solved by cryo-electron microscopy PDB 6I2T and EMD-0256 (Leung et al., 2018; Boyko et al., 2019) (Figures 1A,B).

The tetramer is a dimer of dimers in which monomeric subunits are linked by a single disulfide bond. The 4 subunits tightly interact at each C-terminus through a tryptophan-rich four-helix bundle (tetramerization domain) embedding a short polyproline-rich peptide. The monomer of BChE is a single domain protein (Figure 1B) with an α/β fold similar to AChE, and the active site serine (S198) is located at the bottom of a deep active site gorge (Figure 1C) as for AChE monomer (Sussman et al., 1991). Yet, ChEs are very active enzymes, displaying a high turnover with acetylcholine: kcat of human BChE for acetylcholine is 33,000 min^−1^. This value is about 10 times smaller than kcat of AChE for this substrate (Mukhametgalieva et al., 2022). However, while AChE is inhibited by excess of positively charged substrates such as acetylcholine, BChE is activated. Despite decades of investigations, the underlying mechanism behind these opposing behaviors is still being elucidated (Mukhametgalieva et al., 2023). Thus, the presence of BChE in cholinergic synapses and neuromuscular junctions close to AChE plays a role in a defense mechanism against rapid high increase in acetylcholine concentration (Reed et al., 2010). Also, studies with KO mice for AChE showed that neuronal BChE can act as a surrogate AChE (Duysen et al., 2009; Mesulam et al., 2002), allowing the mice to live despite the complete absence of AChE.

**FIGURE 2:**
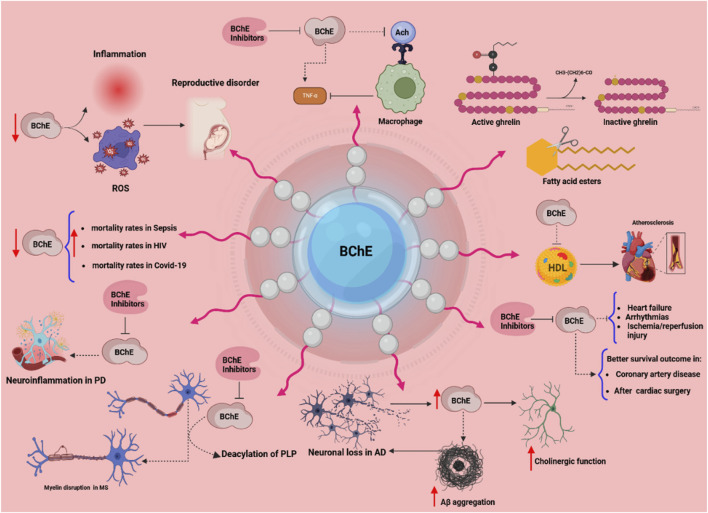
The multifaceted physiological and pathological roles of butyrylcholinesterase (BChE). This scheme illustrates diverse functions of BChE across various systems. Reduced BChE activity linked to inflammation and oxidative stress (ROS) contributes to reproductive disorders, immune activation, and elevated mortality in conditions such as sepsis, HIV, and COVID-19. In the nervous system, in addition to its role in the cholinergic system, BChE plays roles in neuroinflammation (Parkinson’s disease), myelin disruption (multiple sclerosis), and neurodegeneration (Alzheimer’s disease) via mechanisms involving PLP deacylation and Aβ aggregation. BChE regulates macrophage activation by hydrolyzing acetylcholine (ACh), and its inhibition may suppress TNF-α release. In lipid metabolism, BChE hydrolyzes fatty acid esters and inactivates ghrelin, implicating it in energy regulation. Cardiovascularly, BChE correlates with HDL levels and impacts outcomes in heart failure, arrhythmia, and ischemia/reperfusion injury. Clinical data suggest that higher BChE activity is associated with better survival in coronary artery disease and post-cardiac surgery. BChE inhibitors are depicted as modulators that may attenuate pathological processes. Arrows indicate increased (↑) or decreased (↓) activity; dotted lines denote direct interactions.

BChE hydrolyzes acetylcholine and multiple types of neutral and positively charged esters (carboxylic-, phosphoric-, carbamyl-), and arylacylamides of pharmacological and toxicological interest (Masson et al., 2023). The complexity of catalytic mechanisms with certain substrates has been reviewed and recently expanded (Mukhametgalieva et al., 2023; Reed et al., 2010; Masson et al., 2023; Masson and Lockridge, 2010; Mukhametgalieva et al., 2024; Masson, 2012). However, it is not yet known whether the catalytic complexities observed, such as hysteretic behavior and slow-binding reactions with medicinal and toxic substrates are physiologically or pharmaco-toxicologically relevant. The discovery of several natural substrates such as the hunger hormone ghrelin (De Vriese et al., 2004) and long-chain fatty acid esters that are degraded by BChE (Kinchen et al., 2021) led to the emergence of new physiological functions for BChE (Gok et al., 2024). However, the regulation of BChE expression by microRNAs and the polyallelism of BChE contribute to complexify the biology of this enzyme. This review would like to clarify novel aspects about the physio-pathology and pharmacology of BChE, with a special attention to those resulting from the post-transcriptional control of BChE expression by microRNAs. These new developments were missing in the most recent reviews about this enzyme (Lockridge, 2015). Thus, in addition to basic knowledge about this enzyme, the most relevant and recent advances in the physiological and pharmaco-toxicological involvements of BChE are reviewed. The following scheme (Figure 2) summarizes all currently known implications of BChE in physio-pathological and toxico-pharmacological processes. These implications are developed in the following sections.

## 2 Pharmaco-toxicological roles of BChE: drug and toxicant metabolism

This importance of BChE was first recognized for its implication in metabolism of certain carboxyl-ester-containing drugs and as a target of different poisonous carbamyl-and phosphyl-esters. We recently reviewed the mechanistic aspects of BChE-catalyzed reactions with these esters and role of BChE in pharmacology and toxicology (Masson et al., 2023). BChE is also reversibly inhibited by various natural and artificial chemicals. Here we will only highlight the most significant aspects of these reactions/interactions from a public health perspective.

BChE is mostly found active in tissues of first contact, including the skin, plasma, liver, lungs, and tissues that are places for interaction with substances entering the body (Šisková et al., 2012). In plasma and liver, BChE plays a substantial role in ester-containing drug metabolism. BChE also converts ester-prodrugs into their active pharmacological forms and inactivates other drugs by degrading them. Its metabolic efficiency and consequently drug effectiveness and clearance, can significantly be affected by genetic mutations and structural variations (Sridhar and Gumpeny, 2024).

### 2.1 BChE in metabolism of medicinal esters and detoxification of poisonous esters

Hydrolysis of carboxyl-esters - BChE has a prominent role in metabolism of numerous ester-containing drugs and also arylacylamide compounds (Masson et al., 2023). However, at this point, it must be mentioned that BChE is a promiscuous enzyme. Thus, it cannot be ruled out that BChE can also hydrolyze other compounds such as physiologically and pharmacologically relevant lactones and thiolactones. A few significant examples of BChE-catalyzed ester degradation will be mentioned. In particular, curarisants such as succinylcholine and mivacurium, neuromuscular blockers are degraded by BChE in the bloodstream, facilitating their clearance and preventing their side effects. However, deficiencies or mutations in BChE can result in prolonged apnea and delayed recovery from curarisant-involved anesthesia, showing its key role in patient safety (Sridhar and Gumpeny, 2024). Although BChE hydrolyzes numerous toxic carboxyl-esters used as medical drugs or narcotics (Masson et al., 2023), cocaine as a major narcotic is a worldwide public health concern. Cocaine, a stimulant extracted from the leaves of Erythroxylon coca, has sympathomimetic properties, mimicking the effects of the sympathetic nervous system. Cocaine addiction poses a significant challenge to public health and medical practice due to its widespread abuse and associated health risks (Inaba, 1989). Cocaine is primarily metabolized through hydrolysis, a process that can occur spontaneously or via enzymatic activity. BChE is a key enzyme in this process, converting cocaine into a less active compound, ecgonine methyl ester (EME). Additionally, liver carboxylesterases metabolize cocaine into two products: benzoylecgonine (BE) and EME. In addition, liver cytochrome P450 (CYP450) enzymes process approximately 5% of cocaine into norcocaine, a pharmacologically active and highly hepatotoxic metabolite. Importantly, BChE further breaks down norcocaine into a non-toxic product known as norecgonine methyl ester (DEME), highlighting its role in mitigating cocaine toxicity (Xie et al., 1999). BChE exhibits a strong stereospecificity for cocaine enantiomers. It is approximately 2,000 times less efficient at hydrolyzing the naturally occurring (−)-cocaine compared to its synthetic enantiomer, (+)-cocaine (Gatley, 1991). Due to this limited catalytic efficiency, researchers have engineered mutants of human BChE, collectively termed “cocaine hydrolases” (CocH), to enhance its activity against naturally-occurring cocaine. The first one was the mutant A328Y (Xie et al., 1999). Next-generation of mutants, CocH1, CocH3 and other BChE mutants, with progressively improved catalytic efficiency and therapeutic potential were made by combining computer-design and directed evolution approaches (Gao et al., 2006). Thus, BChE plays a vital role in detoxifying cocaine by converting it into non-toxic or less toxic metabolites. While the natural enzyme efficiency is limited, engineered BChE mutants offer promising applications in treating cocaine overdose and addiction (Sun et al., 2024). At this point, we must mention that a new designed BChE mutant capable of degrading cocaine was also found to react with paraoxon and be able to rescue mice poisoned with a lethal dose of this OP and accelerate the recovery of poisoned animals (LeSaint et al., 2025).

Organophosphorus and carbamate poisoning–In addition to reaction with various poisonous carboxyl-esters, BChE reacts with toxics phosphoryl-esters (OPs), carbamyl-esters and plays a role in neutralization and metabolism of these compounds. OPs and carbamates are mostly used as pesticides but the most potent OPs are banned chemical warfare agents. The prevalent use of OP pesticides increases the risk of accidental, occupational, or deliberate acute poisoning (Chowdhary et al., 2014; Hajimohammadi et al., 2024). OPs inhibit AChE and BChE by phosphylating a key serine residue in the enzyme active center (S198 in human BChE) that prevents it from breaking down ACh, leading to excessive ACh accumulation in synaptic clefts. The overactivation of muscarinic and nicotinic receptors causes symptoms of cholinergic toxicity. Moreover, AChE and BChE following inhibition by OP, undergo a process called “aging,” where the bound OP molecule undergoes further progressive chemical changes (dealkylation), making reactivation of phosphylated enzymes even impossible. In most cases, the natural recovery of AChE activity by water-mediated reactivation is too slow to counter the effects of OP toxicity (Worek et al., 2016). In that respect, BChE can be regarded as a protective enzyme of AChE against OPs. Therefore, administration of purified BChE has been used as a protective agent (bioscavenger) against OP poisoning. BChE binds and reacts with OP molecules, neutralizing them before they can inhibit synaptic AChEs. This turns the irreversible inhibition of BChE from a disadvantage into a benefit, as it protects AChE and other biological targets (Doctor and Saxena, 2005). BChE and its engineered forms are classified into three types of bioscavengers based on their mechanism of action: stoichiometric bioscavengers, catalytic bioscavengers, and pseudo-catalytic bioscavengers. The first ones bind OP molecules in a molar ratio (1:1). It requires a large amount of enzyme, which makes it very expensive and less practical for therapy. The second ones are BChE mutants engineered to break down OPs rapidly through enzymatic hydrolysis. They are effective in small quantities, even against lethal doses of OP. The last ones are a combination of BChE and oxime reactivators. Oximes restore the activity of BChE after it binds the OP adduct and reactivates the enzyme (Nachon et al., 2013; Xing et al., 2021). This creates a pseudo-catalytic turnover where BChE can continuously neutralize OP molecules. New click-chemistry-derived oximes with fast reactivation rates of OP-inhibited BChE makes this approach promising (Čadež et al., 2025). Although, engineering of BChE to make pseudo-catalytic and catalytic bioscavengers led to too slow mutated enzymes (Lushchekina et al., 2018), new QM/MM approaches for *in silico* investigation of catalytic mechanisms and computer-modeling of novel enzyme give hope for the imminent creation of BChE mutants capable of degrading OPs at high rate (Blinova et al., 2024; Zlobin et al., 2024).

Altogether, BChE mediates the clearance of OP in various ways and plays an important role in preventing poisoning by *in vivo* detoxification. Carbamate pesticides are also significantly toxic for humans and animals (Kumar et al., 2022). BChE, transiently carbamylated by these compounds can, thus, reduce their harmful effects by neutralizing them in the bloodstream before they inhibit synaptic AChE (Sridhar and Gumpeny, 2024). At this point, it is interesting to note that the current prophylactic treatment of OP poisoning is based on the use of carbamates (pyridostigmine or physostigmine) that transiently protect the active site of AChE against reactive OPs (Lorke and Petroianu, 2019).

Finally, because BChE forms covalent (permanent or transient) adducts with OPs and carbamates, mass spectrometry analysis of proteolytic BChE adduct fragments is a retrospective marker tool of exposure to these compounds and allows identification of the parent toxicants responsible for poisoning (Schopfer and Lockridge, 2012; Van der Schans et al., 2013; Ren et al., 2023).

Reversible binding of toxic compounds to BChE - Many chemicals bind to BChE and act as reversible inhibitors. Some are natural compounds, while others are xenobiotics or drugs used for therapy of various diseases. Here we will limit to two chemicals that are of public health concern:

Glycoalkaloids - Some plants including potatoes and tomatoes produce glycoalkaloids (solanine and chaconine), that can be toxic if consumed in high amounts. These compounds inhibit BChE, and potentially interfere with BChE-catalyzed metabolism of certain drugs, thus, contributing to their toxic effects (McGehee et al., 2000). In addition, inhibition of BChE by glycoalkaloids present in food might lead to misdiagnosis of OP poisoning (Nigg et al., 1996).

Fluoride - The reversible inhibiting action of fluoride ions (uncompetitive inhibition) on BChE has long been known (Cimasoni, 1966; Masson et al., 1993), and was even used for phenotyping BChE variants (Harris and Whittaker, 1961). Several studies evaluated genetic variations in BChE for sensitivity to fluoride toxicity from groundwater exposure. A Study conducted in Pakistan, demonstrated elevated BChE activity in clinically healthy adults with fluorosis, a condition caused by excessive fluoride intake. These results shows that difference in BChE levels is associated with the body’s response to fluoride toxicity (Bibi et al., 2023).

## 3 New physiological roles of butyrylcholinesterase (BChE)

Traditionally, BChE was considered as a complementary or secondary enzyme to AChE, mainly involved in the hydrolysis of acetylcholine in plasma and peripheral tissues. This point of view originated from higher turnover of AChE for acetylcholine and its priority in cholinergic neurotransmission in synaptic clefts, which appeared to shadow the significance of BChE (Masson et al., 2023). Moreover, BChE was viewed as non-essential, based on studies showing normal development in BChE-deficient animals (Li et al., 2008a; Li et al., 2008b) and the overall wellbeing of individuals who lack BChE activity, the “silent” phenotypes (Mano et al., 2007). However, recent research has significantly increased our knowledge about BChE functions. Beyond its role as an enzyme that necessarily completes and/or surrogates the essential role of AChE in the cholinergic system (in particular to protect AChE against low doses of OPs), BChE has been implicated in various physiological and pathological processes. These include detoxification of various toxic esters, drug metabolism, lipid regulation, modulation of immune and cardiovascular systems, and a potential role in neurodegenerative diseases such as Alzheimer, Parkinson, and multiple sclerosis (Sun et al., 2024). This evidence redefined BChE as a promiscuous and versatile enzyme with critical roles, shifting its scientific perception from a backup enzyme in the cholinergic system to a key enzyme in a variety of biological systems. Emerging roles of BChE in non-nervous system tissues and in disease states as well as implication of miRNA regulation of BChE in multiples physio-pathological processes are developed in this section.

**FIGURE 3:**
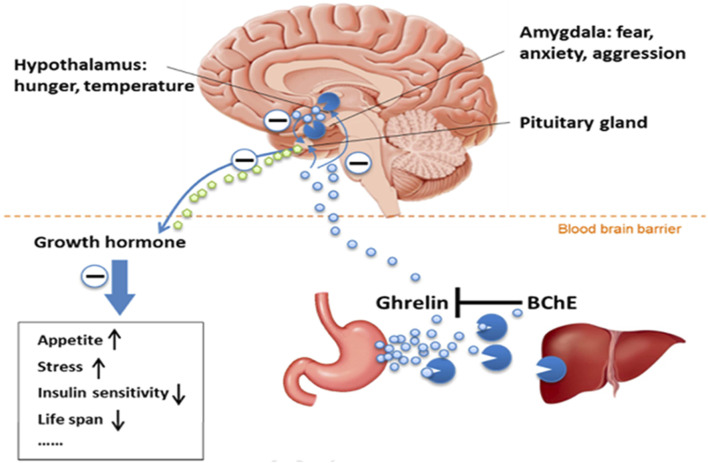
View of the BChE-ghrelin axis (Brimijoin et al., 2016) (Reproduced with permission of Elsevier). Acylated ghrelin, the hunger hormone is predominantly synthesized in the stomach and released in the blood stream. This hormone is also synthesized in hypothalamus and amygdala. Acylated ghrelin acts in the brain in pituitary gland, hypothalamus and amygdala. Its main action is in pituitary gland where it stimulates the growth hormone release. Acylated ghrelin is deacylated (inactivated) by BChE in plasma and liver.

BChE And Lipid Metabolism - Among side functions, BChE has long been considered to be involved in the metabolism of fatty acids. Indeed, BChE plays a critical role in lipid metabolism and then maintaining energy homeostasis through hydrolyzing lipid-based compounds and an acetylated peptide, ghrelin, involved in lipid metabolism. Ghrelin, is a hormone, the “hunger” hormone that contributes in appetite regulation, lipid metabolism, and energy balance (Brimijoin et al., 2016). This hormone mainly synthesized in the stomach and several areas of the brain exists in two forms, acylated ghrelin, and deacylated ghrelin. Acylated ghrelin is the active form that binds to growth hormone secretagogue receptor 1a (GHSR1a) to promote growth hormone secretion and regulate lipids. BChE inactivates ghrelin through deacylation, by removing its octanoyl group, leading to reduction in appetite and reduce lipid profile (Figure 3). This shows the significant role of BChE in maintaining energy homeostasis and reducing obesity risk (Shi et al., 2017; Amir et al., 2025).

In addition to ghrelin, BChE breaks down fatty acid esters such as arachidonoylcholine, and 4-methylumbelliferyl palmitate, modulating lipid signaling in the liver, adipose tissues, and bloodstream. Previous study indicates that essential fatty acids, including alpha-linolenic acid (ALA) and linoleic acid (LA), can regulate BChE expression, affecting lipid homeostasis (Shi et al., 2017). In particular, the reversible inhibitory action on BChE of released unsaturated fatty acids suggests also a possible allosteric regulation of the BChE activity (Akay et al., 2025; Gok et al., 2025).The authors of these works point out several potential positive physiological effects of this inhibition in lipid metabolism and in attenuation of cognitive decline in AD. However, the role of BChE in metabolic health is context-dependent. While moderate BChE activity supports lipid metabolism and energy balance, elevated BChE levels are associated with pathological conditions. For instance, in metabolic syndrome (MetS) and type 2 diabetes (T2D), increased BChE levels correlate with markers such as leptin, glucose, cholesterol, inflammatory mediators like interleukin-6 (IL-6), and oxidative stress indicators such as superoxide anion. These findings suggest that while BChE is a biomarker of metabolic disorders, its elevated activity may contribute to disease progression (Gok et al., 2024; Shi et al., 2017; Villed et al., 2024). In hypercholesterolemic patients, increased BChE levels are linked to decreased high-density lipoprotein (HDL) levels, an unfavorable association given HDL protective role against atherosclerosis. Similarly, in nonalcoholic fatty liver disease (NAFLD), the ratio of BChE to HDL correlates with disease severity and risk of atherosclerosis (Sun et al., 2024).

BChE And Inflammation Regulation - The cholinergic system plays a critical role in the regulation of inflammation through the cholinergic anti-inflammatory pathway. In this pathway, ACh acts as a key signaling molecule that binds to α7 nicotinic acetylcholine receptors (α7 nAChR) present on the membranes of immune cells, such as macrophages. This interaction reduces the production of pro-inflammatory cytokines, including tumor necrosis factor-alpha (TNF-α), and helps to maintain immune homeostasis. BChE, indirectly regulates this pathway. Indeed, BChE by breaking down ACh inhibits anti-inflammatory effects of cholinergic signaling (Ofek et al., 2007; Pavlov et al., 2003). Certain BChE inhibitors, have emerged as promising therapeutic agents by elevating ACh levels, enhancing α7 nAChR activation, and reducing systemic inflammation. Two notable classes of MTLs, 2-[4-(4-substitutedpiperazin-1-yl) phenyl] benzimidazole derivatives and 2-(hydroxyl-(2-nitrophenyl) methyl) cyclopentanone (2NCP) exhibit significant BChE inhibitory activity with additional neuroprotective effects (Ullah et al., 2021; Ozadali-Sari et al., 2017).

The benzimidazole derivatives (3b, 3) have been recognized as selective BChE inhibitors with IC50 values of 5.18 and 5.22 μM, respectively. In addition to BChE inhibition, these compounds display Aβ anti-aggregation and neuroprotective effects, making them promising agents for neurodegenerative and inflammatory conditions. Their MTL nature suggests they have therapeutic potential beyond Alzheimer disease, especially in inflammatory disorders (Ozadali-Sari et al., 2017). Furthermore, 2NCP has been demonstrated to substantially lower both AChE and BChE activity, with IC50 values of 17 μg/mL and 23 μg/mL, respectively. Beyond cholinesterase inhibition, 2NCP shows potent antioxidant activity, markedly decreasing oxidative stress markers such as glutathione level in blood (GSH) and catalase. In addition, 2NCP downregulates pro-inflammatory cytokines (IL-1β, IL-6, TNF-α) while increasing the antioxidant transcription factor Nrf2, leading to exerting broad anti-inflammatory and neuroprotective effects. *In vivo* studies in 5xFAD mice further showed that 2NCP treatment led to cognitive and motor improvements, highlighting its effectiveness on therapeutic targets (Ullah et al., 2021).

The multitarget nature of these BChE inhibitors shows their wide physiological role in modulating inflammation, oxidative stress, and neurodegeneration. Moreover, recent studies, such as Khan et al. (2023), reported that BChE activity, markedly reduced in workers exposed to xenobiotics, is linked to increased pro-inflammatory cytokines levels (Khan et al., 2023). This highlights the systemic role of BChE in inflammation regulation and offers that targeting BChE could be a promising way for inhibiting inflammation-related disorders. Finally, this evidence shows that BChE inhibitors like benzimidazole derivatives (3b, 3) and 2NCP have great ability as multitarget therapeutics. By modulating cholinergic signaling, oxidative stress, and inflammatory pathways, these molecules express a promising avenue for the development of novel treatments for neurodegenerative diseases and inflammatory disorders.

BChE And Cardiovascular System - BChE is involved in both the normal function of the heart and heart disorders, specially through its roles in inflammatory regulation, neural regulation, and lipid metabolism. BChE is expressed in different parts of the heart, including cardiomyocytes, and is especially prominent in the ventricles, where its activity surpasses that of AChE. The presence of BChE in the ventricular system shows its activity in the non-neuronal cholinergic system (Dingová et al., 2025; Kilianova et al., 2020). Research that evaluated the effects of BChE inhibitors on the heart indicated its cardioprotective action, highlighting a role for BChE in maintaining cardiac homeostasis and response to pathological conditions (Hsiao et al., 2021; Turecký et al., 2021). By hydrolyzing acetylcholine, BChE plays a role in the heart non-neuronal cholinergic system and collaborates with the neuronal system to balance sympathetic activity. Studies on mutant mice deficient in BChE showed its importance in regulating heart rate and contractility, especially under stress conditions. Cholinesterase inhibitors show a therapeutic potential for treatment of disorders such as heart failure, arrhythmias, and ischemia/reperfusion injury, acting by increasing anti-inflammatory responses, enhancing angiogenesis, and reducing cardiac remodeling. Recent insights suggest that BChE presence in the endoplasmic reticulum of ventricular cardiomyocytes may be important for producing and assembling its active forms. This function plays a key role in mitigating pathological conditions, highlighting its importance in cardiac resilience and adaptability. The unique localization and distribution of BChE in heart emphasize its potential as a promising therapeutic target for cardiovascular diseases (Dingová et al., 2025).

NAFLD-Related Cardiovascular Risk - Nonalcoholic fatty liver disease (NAFLD) commonly co-occurs with cardiovascular diseases (CVDs). NAFLD patients often exhibit elevated BChE activity in plasma and reduced HDL cholesterol levels, developing an unsuitable lipid profile. The BChE/HDL ratio, also referred to as the Kutty index, is a novel marker for cardiovascular risk. A higher Kutty index is associated with greater severity of atherosclerosis and increased cardiovascular events. This evidence emphasizes the dual role of BChE as a biomarker for cardiovascular risk and a therapeutic target in treating metabolic and cardiovascular diseases (Harris and Whittaker, 1961).

Coronary Artery Disease (CAD) - Evaluating BChE activity in serum provides valuable perspective into disease severity and prognosis in coronary artery disease (CAD), especially acute coronary syndrome (ACS). It has been shown that patients with acute myocardial infarction (AMI) exhibit substantially lower BChE activity compared to healthy individuals (Kocabaş et al., 2016). In addition, higher BChE levels are associated with better survival outcomes, particularly in ACS patients aged 45–65. This inverse relationship between adverse cardiovascular outcomes and BChE levels suggests determination of BChE activity in plasma as a risk factor and prognostic biomarker in CAD (Sulzgruber et al., 2015).

Post-Surgical Cardiovascular Management - Plasma BChE activity is also significant in the context of acute cardiovascular conditions. For instance, in patients receiving veno-arterial extracorporeal membrane oxygenation (VA-ECMO) treatment after cardiac surgery, BChE activity level was associated with survival outcomes. These findings further highlight the significance of enzyme level in acute and chronic cardiovascular disorders (Distelmaier et al., 2014).

### 3.1 BChE and neurological functions and neuropathology

Alzheimer Disease (AD) - As mentioned before, BChE complements AChE activity in the central nervous system by breaking down ACh and maintaining cholinergic signaling. This role becomes particularly significant in Alzheimer disease (AD), where AChE levels decline due to neuronal loss. BChE compensates for the reduced AChE activity by increasing its own activity (Greig et al., 2001). This suggests that the compensatory mechanism in AD is increased neuronal biosynthesis of BChE. While this compensatory mechanism helps to sustain cholinergic function, it also promotes amyloid-beta (Aβ) aggregation, thus, contributing to neurotoxic plaque formation. Histochemical studies identified BChE within Aβ plaques in the hippocampus and cortex, establishing its potential as both a biomarker and a therapeutic target in AD (Gok et al., 2022).

The cholinergic hypothesis is further supported by the effectiveness of cholinesterase inhibitors in managing AD symptoms (Sun et al., 2024). However, genetic variants of BChE, in particular the K-variant (A539T), may increase AD risk by impairing BChE function (Wang et al., 2015). This variant reduces ACh hydrolysis, leading to ACh accumulation in CNS, causing dysregulated cholinergic signaling, and cognitive dysfunction. Additionally, chronic cholinergic imbalance exacerbates neuroinflammation and stress, indirectly contributing to AD pathology (Gok et al., 2022). The K-variant may also have a synergic effect with other risk factors, including the ε4 allele of apolipoprotein E (APOE ε4), that is a well-documented genetic contributor to AD (Ratis et al., 2023). This evidence highlights the complex role of BChE in AD and its ability as a therapeutic target to manage cholinergic imbalances and protein aggregation (Zhou and Huang, 2022).

Multiple Sclerosis (MS) - BChE plays a significant role in multiple sclerosis (MS) through deacylation of proteolipid protein (PLP) a main component of myelin. BChE-catalyzed deacylation of PLP disrupts the stability of myelin and enhances neuroinflammation, a hallmark of MS (Darv et al., 2010). Certain cholinesterase inhibitors, such as donepezil (Aricept) used in the palliative treatment of AD, showed significant reduction in inflammation, lymphocyte proliferation, and pro-inflammatory cytokine secretion. These effects help to maintain myelin structure, offering potential therapeutic benefits against MS (Christodoulou et al., 2006; Nizri et al., 2006).

Parkinson Disease Dementia (PDD) - BChE is involved in Parkinson disease dementia (PDD). Inhibiting BChE can increase ACh concentration, improving cholinergic signaling and reducing neuroinflammation. This suggests that BChE inhibitors could potentially be used in managing PDD symptoms (Villed et al., 2024).

Stroke and Ischemic Brain Injury - Lower activity of plasma BChE has been observed in patients with ischemic stroke compared to healthy individuals, likely due to the brain response to injury or systemic inflammation. Reduced BChE levels during or immediately after a stroke may provide insight into the severity of the vascular event and a helpful guide to prognosis (Vaisi-Raygani et al., 2009).

Traumatic Brain Injury (TBI) - BChE levels may serve as a marker of injury, severity and predict adverse outcomes, including mortality in traumatic brain injury (TBI). It was shown that non-survivors from TBI, have significantly lower plasma BChE activity than survivors. Thus, BChE activity could potentially be used as a biomarker for TBI severity and prognosis (Zhang et al., 2015).

Butyrylcholinesterase and Hyperhomocysteinemia: Interaction with Homocysteine Thiolactone - BChE appears to play a complex role in the context of elevated homocysteine and its toxic metabolite, homocysteine thiolactone (Hcy-thiolactone), which is increasingly recognized as a contributor to cardiovascular and neurological disorders. Unlike AChE, which is slowly and irreversibly inhibited by Hcy-thiolactone, BChE activity is significantly enhanced in its presence, likely due to specific structural interactions involving the unprotonated amino group of the thiolactone (Darvesh et al., 2007). This activation of BChE may represent a compensatory mechanism in hyperhomocysteinemic states, although its physiological consequences remain to be fully elucidated. Clinical studies further support the association between increased BChE activity and untreated cystathionine β-synthase (CBS) deficiency, a condition characterized by high homocysteine levels, oxidative stress, and inflammation suggesting that BChE may serve as a biomarker or mediator of metabolic disturbances in these patients (Vanzin et al., 2015). Mechanistically, Hcy-thiolactone contributes to vascular and neurological damage by modifying lysine residues on proteins, leading to immune responses and protein dysfunction, highlighting the toxic potential of this metabolite in various pathologies (Chubarov, 2021). Taken together, these findings suggest that BChE is not merely a passive player but may actively respond to or influence the pathogenic landscape shaped by homocysteine metabolism.

### 3.2 BChE and infection/sepsis

Sepsis - Sepsis is a severe condition marked by acute organ dysfunction caused by an abnormal immune response to infection. It is one of the major causes of mortality in intensive care units. Research shows that lower plasma BChE levels correlates with higher mortality rates in sepsis patients. Notably, patients who died within 90 days of admission exhibited significantly lower BChE activity compared to survivors (Zivkovic et al., 2018). Measuring BChE levels at admission may serve as a useful prognostic tool, align with other clinical evaluations in critical care units. In patients diagnosed with sepsis-3, lower BChE levels was identified as an independent risk factor for 30-day mortality. These findings offer that BChE activity not only indicates the severity of sepsis but could also help treatment decisions and prognosis evaluations (Peng et al., 2018).

HIV - A study investigating the 6-month outcomes of patients receiving highly active antiretroviral therapy (HAART) for HIV infection found that low BChE levels in plasma were linked to poorer survival. Patients with decreased BChE levels had a 1-year survival rate of (64.5% ± 4.5%), while those with normal BChE levels had a significantly higher rate of survival (87.6% ± 1.8%). This suggests that plasma BChE activity is a potential marker in predicting survival outcomes and assessing the efficacy of HAART in HIV-infected individuals (Xu et al., 2018).

Hansen’s Disease (Leprosy) - Genetic studies showed that the frequency of the 70G allele and the D70G genotype of BChE (“atypical” phenotype) is higher in patients with Hansen disease (leprosy). Atypical BChE variants by interfering with the inflammatory response, increase susceptibility to infection, having compromised the body’s natural defense against infection (Gomes et al., 2012). These findings offer a potential connection between BChE genetic polymorphisms and the risk of developing Hansen disease.

Foot and Mouth Disease (FMD) - Increased BChE levels has been observed in cases of Foot and Mouth Disease (FMD) induced by enterovirus 71 infection in children. This finding demonstrates that BChE may be involved in the immune response to viral infections, especially influencing disease progression and recovery (Cheng et al., 2016).

COVID-19 - The role of BChE has been evaluated as a prognostic tool in critically ill COVID-19 patients. Findings reveal that reduced plasma BChE levels correlate with increased disease severity, organ dysfunction, and higher risk of mortality rates (Espeter et al., 2022). Continuous decline in BChE activity, especially in non-survivors, demonstrates its ability as an early biomarker for poor clinical outcomes. Moreover, plasma BChE levels have an inverse relation with common inflammatory markers such as C-reactive protein (CRP) and interleukin-6 (IL-6), emphasizing its connection to systemic inflammation. Point-of-care testing (POCT) of BChE could facilitate early risk assessment, supporting ICU admission decisions and treatment planning in high-risk COVID-19 patients (Markuskova et al., 2023).

### 3.3 BChE and reproductive health

Fertility - There is a connection between BChE activity and reproductive health, especially in women suffering from idiopathic unexplained infertility (infertility with no identifiable cause). Measurement of plasma BChE levels 1 day before and 1 day after intra-uterine insemination (IUI) procedures demonstrated a positive correlation between BChE levels and total antioxidant activity on the day before the procedure. BChE levels improve antioxidant defense, potentially inhibiting oxidative stress, a factor known to impair fertility, in reproductive cells. These findings suggest that BChE could play a positive role in the reproductive system by improving fertility outcomes (Sridhar and Gumpeny, 2024).

Pre-eclampsia - Another serious pregnancy-related condition is pre-eclampsia, characterized by hypertension and proteinuria. BChE activity is substantially lower in women suffering from pre-eclampsia than those with normal pregnancies. This may reflect BChE contribution in pathophysiology of pre-eclampsia, as lower enzyme activity is connected to oxidative stress, inflammation, or other maternal health changes related to pre-eclampsia (Rahimi et al., 2013). These results suggest that reduced BChE levels could be used as a beneficial biomarker for early identification or monitoring of pre-eclampsia during pregnancy (Sridhar and Gumpeny, 2024).

BChE and Liver Function - Plasma BChE is produced in hepatocytes. It can be used as a reliable biomarker for monitoring liver function. Alteration in BChE plasma levels reflects the capacity of the liver to synthesize proteins, providing a good view in liver health and disease progression (Sridhar and Gumpeny, 2024). In acute hepatitis, higher inflammation levels affect the liver ability to synthesize necessary enzymes, resulting in reduced circulating BChE. Measuring these levels can help with identification and diagnosis of the extent of liver dysfunction and monitoring recovery during treatment (Sridhar and Gumpeny, 2024). In cirrhosis, a chronic liver conditions, the liver synthetic capacity substantially decreases, leading to significantly low BChE activity. This decline is associated with disease severity, making BChE an important marker for staging cirrhosis and helping therapeutic management strategies (Pohanka, 2013).

BChE and Nutritional Status - BChE is used as a potential biomarker for evaluating nutritional status, especially in persons suffering from malnutrition or protein deficiencies. In malnourished children, especially those with visceral undernutrition, low BChE levels are often observed. This decline indicates the lack of protein availability and total nutrient deficiency, reflecting the body’s compromised metabolic and biosynthetic functions (Dabke et al., 1972). In adults, BChE assessment is beneficial for identifying nutritional risks. Low BChE activity is related to protein-energy malnutrition, a condition common in aging individuals that disposes them to further health problems. Early recognition with measuring BChE levels can guide dietary and medical interventions to improve outcomes (Santarpia et al., 2013).

## 4 MicroRNA regulation of BChE

MicroRNAs (miRNAs) are small RNA molecules, typically 21–25 nucleotides in length, that play an essential role in regulation of gene expression. These molecules have attracted attention as therapeutic targets and potential biomarkers for various diseases. miRNAs bind to complementary sequences in the 3-untranslated region (3-UTR) of target mRNAs, a process mediated by the RNA-induced silencing complex (RISC). This interaction between miRNAs and mRNAs leads to suppression of protein translation, which reduces biosynthesis of protein, or cleavage of mRNAs (Bartel, 2004). One of the most significant features of miRNAs are their network-like activity, where one miRNA can target multiple mRNAs, and a single mRNA can be regulated by several miRNAs. This complicated relationship highlights that miRNA are multifunctional and control different biological processes. miRNAs levels can be modulated, using specific molecules. Antago-miRs are designed to suppress specific miRNAs while synthetic mimics are developed to increase miRNA activity (Madrer and Soreq, 2020). Relations between miRNAs and BChE, and their consequences and implications in physio-pathology, pharmacology and toxicology are summarized in Table 1.

**TABLE 1:** miR and BChE, physio-pathological impact and therapeutic implications.

miRNA	Target/Interaction with BChE	Molecular mechanisms	Physiological/Pathological impact	Clinical evidence	Therapeutic implications	Administration strategy	Ref.
miR-186	Predicted to target BChE mRNA, potentially suppressing translation; paradoxically, stress increases BChE activity	Forms a feedback loop in stress responses; BChE activity may be regulated via compensatory mechanisms, post-translational modifications, or alternative transcriptional regulation	Modulates BChE and ACh levels during stress; regulates inflammation and stress responses	In an experimental model (e.g., predator scent stress in C57/B6J mice), miR-186 levels and BChE activity increased in intestinal tissues, in response to stress Plasma BChE levels have been linked to improved outcomes in stroke	Potential role in balancing ACh signaling for neuroprotection; may influence neuroinflammatory and cognitive processes	miR-186 mimics (synthetic oligonucleotides) could be administered to increase BChE production and modulate stress/inflammation	Nadorp and Soreq (2014)
	Regulates BChE production; targets feedback mechanisms limiting BChE secretion	Enhances BChE secretion from HUCPVCs by interrupting innate inhibitory pathways	Supports increased BChE biosynthesis; potential use in counteracting OP toxicity	Genetic manipulation of HUCPVCs and miR-186 mimics have shown promise in experimental models	Potential strategy for boosting endogenous BChE levels as a bioscavenger against OP poisoning	miR-186 oligonucleotide mimics	Braid et al. (2019)
miR-200c	Suppresses BChE expression, potentially via mRNA degradation	miR-200c likely binds to the 3′-UTR of BChE mRNA, leading to its degradation or inhibition of translation	Contributes to neuroinflammation regulation; may influence stress-related disorders	Studies have shown miR-200c may alter BChE levels in stress models; however, clinical evidence linking miR-200c directly to BChE in human diseases is still limited	Targeting miR-200c inhibition could help enhance BChE expression in conditions related to inflammation or neuroinflammation. This may improve therapeutic strategies for stress and immune-related diseases	miR-200c inhibitors (antagomiRs) could be used to restore BChE levels, offering a potential treatment strategy for neuroinflammation or stress	Braid et al. (2019)
miR-199a-5p	Downregulates BChE activity	Binds to 3′-UTR of BChE mRNA, leading to translational repression	Increases ACh levels, leading to cholinergic overstimulation and bladder pain	Upregulated in bladder pain syndrome patients	Inhibiting miR-199a-5p may relieve bladder pain by restoring BChE function	AntagomiR-199a-5p to restore BChE expression	Nadorp and Soreq (2014)
miR-200 miR-429	Suppress BChE translation	Reduce mRNA stability of BChE	Enhances cholinergic anti-inflammatory effects and stress adaptation	Increased in neuropathic pain models and inflammatory states	Therapeutic potential in inflammatory diseases by regulating cholinergic pathways	miR-200b/429 mimetics to modulate inflammation	Nadorp and Soreq (2014)
miR-155	Downregulates BChE during inflammatory response	Targets BChE mRNA, reducing enzyme expression under inflammatory stress	Aggravates systemic inflammation and liver dysfunction in infections	Elevated in patients with infectious diseases like toxoplasmosis	Targeting miR-155–BChE axis could reduce inflammation	AntagomiR-155 to restore BChE activity	El-Sayad et al. (2021)
miR-129-5p	Activated via non-canonical NFκB signaling, suppress BChE activity	miR-129-5p targets both BChE and AChE mRNA, potentially suppressing their expression. Elevated levels of acetylcholine (ACh) activate α7 nAChR on immune cells, leading to inhibition of pro-inflammatory cytokines	miR-129-5p is linked to inflammation, immune responses, and cholinergic signaling. The suppression of BChE may increase ACh levels, activating the cholinergic anti-inflammatory pathway, which reduces inflammation and promotes immune balance	Experimental studies have shown that TLR9 signaling influences miR-129-5p expression, with weak TLR9 agonists (e.g., BL-7040) modulating miR-129-5p levels to enhance cholinergic signaling and reduce inflammation in inflammatory diseases like IBD	Enhancing miR-129-5p could modulate BChE levels, promoting cholinergic anti-inflammatory effects and offering therapeutic avenues for chronic inflammatory diseases. TLR9 agonists like BL-7040 may help regulate miR-129-5p expression	TLR9 agonists (e.g., BL-7040) could be used to upregulate miR-129-5p and regulate BChE expression, offering therapeutic strategies in chronic inflammatory conditions like IBD	Nadorp and Soreq (2015)

In diseases related to dysregulated BChE activity, miRNA-based therapies are emerging as promising interventions (Table 1). These strategies are now being explored in clinical trials for their potential to exactly modulate BChE levels (Hanna et al., 2019). miRNAs that target BChE mRNA are being regarded as biomarkers, especially in conditions such as inflammatory disorders, neurological disorders, and metabolic dysfunction (Bonneau et al., 2019).

Profiling miRNA networks involved in BChE regulation helps researchers gain valuable information about the enzyme’s broader role in main biological pathways, such as lipid metabolism and inflammatory processes. The growing knowledge of miRNA-mediated BChE regulation not only increases our understanding of main mechanisms involved in certain diseases but also provides new avenues for developing diagnostic tools and targeted therapies.

A study conducted by Hanin et al. identified 116 miRs with potential binding sites for BChE, using computational tools and bioinformatic algorithms, highlighting a wide regulatory network specific to this enzyme. Based on this study notable identified miRs include miR-203, miR-218, miR-221, and miR-222, as well as members of the miR-181 family (miR-181a, b, c, d), in addition, miR-494, miR-200b, and miR-200c were also identified as main regulators of BChE. This evidence proposes that miRs play a key role in fine-tuning BChE expression, and consequently, for its function in both physiological and pathological conditions (Hanin and Soreq, 2011).

CholinomiRs Targeting BChE - CholinomiRs are a group of certain microRNAs that regulate genes within the cholinergic pathway. BChE, a crucial enzyme for modulating cholinergic signaling, is targeted by CholinomiRs. It is worth noting that many CholinomiRs are specific to primates. This suggests the importance of CholinomiRs in the development of complex brain functions and behaviors specific to primates. The regulation of BChE by CholinomiRs highlights their role in fine-tuning cholinergic activity and maintaining neurophysiological balance. Dysregulation of CholinomiRs has been implicated in several neurological and non-neurological conditions. Their involvement in these disorders underscores the potential of CholinomiRs as both biomarkers and therapeutic targets for diseases affecting the cholinergic system (Nadorp and Soreq, 2014).

miR-186: A Complex Regulator of BChE and Stress Responses - miR-186 has been implicated in neuroinflammatory processes, stress-related pathologies, and the regulation of BChE. Predicted to target BChE mRNA, miR-186 may suppress its translation and reduce BChE levels under certain conditions. However, recent findings suggest a more nuanced regulatory role, particularly during stress-related responses. In an experimental model, when C57/B6J mice were exposed to predator scent stress, both miR-186 levels and BChE activity were found to increase in their intestinal tissues. This apparent paradox suggests the existence of a complex feedback loop, where stress-induced elevation of miR-186 and BChE activity serves to maintain physiological balance by limiting excessive ACh stimulation. Elevated BChE levels during stress may contribute to anti-inflammatory effects and regulate ACh signaling, supporting recovery processes. For example, higher plasma BChE levels have been associated with improved outcomes in conditions such as stroke (Shenhar-Tsarfaty et al., 2014). Conversely, reduced BChE activity mediated by miR-186 in another conditions leads to decreased ACh breakdown, enhancing cholinergic signaling. This heightened cholinergic activity may amplify anti-inflammatory responses, counteracting stress-induced neuroinflammation. Additionally, prolonged cholinergic activity can enhance synaptic plasticity and memory formation, potentially offering cognitive protection against stress. However, excessive cholinergic activity might also contribute to anxiety or depressive-like behaviors, underscoring the importance of a tightly regulated balance (Nadorp and Soreq, 2014).

Therapeutic Implications of miR-186 and miR-200c in BChE Production - Beyond its role in stress response and inflammation, miR-186 plays a critical role in regulation of BChE production. This may have therapeutic applications. Human umbilical cord perivascular cells (HUCPVCs), that are derived from Wharton’s jelly, secrete BChE naturally. They can be genetically manipulated to increase biosynthesis of BChE. However, there are feedback mechanisms that limit BChE secretion in these cells. Recent findings indicate that miR-186 oligonucleotide mimics can be a promising tool to overcome these inhibitory mechanisms (Braid et al., 2019). This miR-186 can enhance BChE secretion from HUCPVCs, by interrupting the innate feedback mechanisms. This regulatory intervention could lead to more efficient biosynthesis of BChE, which is crucial for implementation of the endogenous enzyme as a stoichiometric bioscavenger against OP poisoning.

In contrast, miR-200c has been identified as a putative repressor of BChE expression. Though its exact mechanism remains unclear, computational predictions suggest that miR-200c may bind to BChE mRNA, leading to its degradation or translational inhibition (Braid et al., 2019). However, experimental studies have not consistently confirmed its role, suggesting that miR-200c′s regulatory effect on BChE may be cell-type specific.

Current approaches, such as the use of plasma-derived BChE, are costly and carry risks (e.g., presence of pathogenic agents or coagulation factors in pharmaceutical enzyme preparations), making miR-186-based modulation as a potentially transformative strategy. Additionally, targeting miR-200c inhibition could further enhance BChE expression, providing an additional strategy to optimize production. Further characterization of miR-186s regulatory network could refine these therapeutic applications, optimizing BChE production for clinical and biopharmaceutical use while providing insights into its broader roles in stress resilience and inflammatory regulation (Braid et al., 2019).

miR-129-5p, miR-186, and miR-200c: Linking Non-Canonical NFκB Pathway to BChE Regulation and Inflammation - Toll-like receptor 9 (TLR9) signaling influences immune responses, including inflammation resolution. Weak TLR9 agonists, such as BL-7040, have been shown to modulate specific miRNAs involved in cholinergic signaling. Among them, miR-129-5p, miR-186, and miR-200c, are predicted to target both BChE and AChE, potentially suppressing their expression. This miRNA-mediated suppression of BChE may enhance cholinergic signaling by decreasing BChE-catalyzed hydrolysis of acetylcholine.

Elevated ACh level activates the α7 nicotinic acetylcholine receptor (α7 nAChR) on immune cells such as macrophages, leading to inhibition of pro-inflammatory cytokines, including TNF-α, IL-6, and IL-1β. This contributes to the cholinergic anti-inflammatory pathway, which attenuates inflammation and promotes immune homeostasis.

The miRNA-mediated regulation of BChE creates a functional bridge between non-canonical NFκB signaling and the cholinergic anti-inflammatory reflex, highlighting its importance in immune regulation. This regulatory mechanism presents a promising significant therapeutic avenue. Weak TLR9 agonists, such as BL-7040, can upregulate miRNAs targeting BChE, offering a novel strategy to modulate inflammation in chronic inflammatory diseases like inflammatory bowel disease (IBD). By enhancing cholinergic signaling, this miRNA-driven regulation could reduce inflammation, and restore immune balance. The interplay between miRNA regulation, non-canonical NFκB signaling, and the cholinergic anti-inflammatory pathway underscores a promising avenue for developing targeted interventions in chronic inflammatory and autoimmune conditions (Nadorp and Soreq, 2015).

miR-199a-5p: A Regulator of BChE in Bladder Pain Pathophysiology - Bioinformatic predictions suggest that miR-199a-5p may bind to the 3′-UTR of BChE mRNA, resulting in the downregulation of BChE activity. This suppression leads to increased ACh accumulation, likely at NMJ of bladder smooth muscles. Increased ACh levels elevate nerve sensitivity, overstimulate muscarinic receptors, and increase neuroinflammation, all of which aggravate bladder pain. While experimental validation is needed, these findings offer the possibility that targeting miR-199a-5p could be a potential therapeutic strategy to reduce cholinergic overactivity and provide relief for patients with bladder pain syndrome and related disorders (Nadorp and Soreq, 2014).

miR-200 Family: Potential Modulators of BChE in Stress and Inflammatory Responses - miR-200b and miR-429, that belong to miR-200 family, play an important role in regulating stress responses and maintaining physiological balance. Both of mentioned miRs are predicted to bind to the 3′-UTR region of BChE mRNA, potentially mitigating its activity. This decreases the hydrolysis of ACh, resulting in elevated ACh levels and enhanced cholinergic signaling, which is vital for modulating stress responses and inflammation regulation. Elevated ACh levels by activating the vagus nerve-mediated anti-inflammatory pathway, attenuate systemic inflammation and oxidative stress while helping recovery from acute stress conditions. Moreover, activated cholinergic signaling affects the hypothalamic-pituitary-adrenal (HPA) axis by suppressing cortisol production that is beneficial during chronic stress. The miR-200 family promotion of cholinergic anti-inflammatory effects underscores its ability in mitigating stress-induced inflammation and oxidative damage. However, excessive downregulation of BChE can lead to cholinergic overstimulation and possibly disruption of autonomic balance. This imbalance may relate to stress-mediated disorders, highlighting a potential regulatory role of miR-200b and miR-429 in maintaining optimal cholinergic signaling and stress adaptation (Nadorp and Soreq, 2014).

miR-155: A Modulator of BChE in Immune Responses and Infections - miR-155 plays a pivotal role in regulating immune responses and has been implicated in the modulation of BChE activity during Toxoplasma gondii infection (El-Sayad et al., 2021). Experimental studies revealed that miR-155 expression is significantly upregulated in infected mice, particularly in response to virulent strains of the parasite, and is inversely correlated with BChE activity. This suggests that miR-155 actively downregulates BChE as part of the inflammatory response to infection.

At the molecular level, T. gondii infection triggers pro-inflammatory cytokine release, including TNF-α, IL-1β, and interferons, which in turn activates the NF-κB signaling pathway. This leads to upregulation of miR-155, amplifying the inflammatory response. A key mechanism by which miR-155 sustains inflammation is through the targeted suppression of Suppressor of Cytokine Signaling 1 (SOCS1), a crucial negative regulator of cytokine signaling. By inhibiting SOCS1, miR-155 prolongs pro-inflammatory cytokine production, contributing to hepatic inflammation and immune dysregulation (Rao et al., 2014).

BChE, having established roles in metabolic regulation and inflammation, exhibits a reduced activity in infected mice, a phenomenon linked to liver tissue damage and dysfunction. The suppression of BChE may result from miR-155s broader regulatory effects on metabolic and immune pathways, further exacerbating cholinergic dysregulation and organ-specific damage. Interestingly, in mice treated with sulfamethoxazole-trimethoprim (SMX-TMP), miR-155 levels gradually return to baseline by day 20 post-infection, allowing BChE activity level to recover (Bottari et al., 2015). This suggests that targeting miR-155 could be a potential therapeutic strategy for restoring BChE function and mitigating liver dysfunction in infectious diseases.

The interplay between miR-155 and BChE highlights a potential mechanism through which inflammation and organ-specific damage are exacerbated during infection. Targeting the miR-155–BChE axis presents an intriguing avenue for therapeutic intervention, particularly in mitigating liver dysfunction and systemic inflammation and liver pathology associated with T. gondii infection (El-Sayad et al., 2021).

## 5 Conclusion

BChE has been recognized as an important enzyme for the metabolism of various drugs and as a target of numerous reversible and irreversible compounds, in particular toxic OPs and carbamates. In this respect, BChE acts as an effective stoichiometric bioscavenger to protect AChE in cholinergic system against irreversible inhibition. However, this approach is very expensive. Thus, enzyme engineering efforts should be pursued to convert BChE to an OP-hydrolyzing bioscavenger, displaying a high turnover. The bioscavenger approach for prophylaxis and treatment of OP poisoning will certainly be improved in the future by associating BChE (wild-type and mutants) to other OP-reacting enzymes (phosphotriesterases) in cocktail formulations. Regarding BChE as a promiscuous enzyme, involved in degradation of several types of toxic chemicals, research on new potential substrates: products of intermediary metabolism, hormones, modulators, drugs and xenobiotics have to be continued. This should stimulate research on novel physiological roles of this enzyme.

Plasma BChE is also a sensitive biomarker that reflects different physio-pathological conditions and poisoning. However, the physiological functions of BChE are still poorly known, though it is established that people homozygous for a silent allele (E_1_^S^E_1_^S^), like the Vysya community of India, do not suffer any physiological troubles under normal conditions (Mano et al., 2007). This contributes to thickening the mystery about the physiological roles of this enzyme in normal conditions. Yet, the control of the expression of the enzyme by miRNA has an impact on multiple physio-pathological processes and pharmaco-toxicological mechanisms. This makes miRNAs as promising treatments for correction of various metabolic dysfunctions and as adjuncts in therapy of OP poisoning. The complex relationship between BChE genetic polymorphism and miRNA-mediated regulation offers a promising area for future research on BChE-catalyzed degradation of drugs. Polyallelism of BChE can alter enzyme activity and stability, which in turn may alter miRNA-mediated regulation and subsequent drug metabolism. This is especially concerning individualized drug responses. Understanding these interactions is expected to provide insights into interindividual variability in drug responses and aid in the development of more personalized therapeutic strategies, ultimately enhancing personalized medicine approaches.

## Abbreviations

ACh, Acetylcholine; AChE, Acetylcholinesterase; AD, Alzheimer disease; BChE, butyrylcholinesterase; OP, organophosphorous compound; Acknowledgments.
